# Novel Therapeutic Approach for the Management of Mood Disorders: In Vivo and In Vitro Effect of a Combination of L-Theanine, *Melissa* *officinalis* L. and *Magnolia officinalis* Rehder & E.H. Wilson

**DOI:** 10.3390/nu12061803

**Published:** 2020-06-17

**Authors:** Vittoria Borgonetti, Paolo Governa, Marco Biagi, Nicoletta Galeotti

**Affiliations:** 1 Department of Neuroscience, Psychology, Drug Research and Child Health (NEUROFARBA), Section of Pharmacology, University of Florence, Viale G. Pieraccini 6, 50139 Florence, Italy; vittoria.borgonetti@unifi.it; 2 Department of Biotechnology, Chemistry and Pharmacy-Department of Excellence 2018-2022, University of Siena, Via Aldo Moro 2, 53100 Siena, Italy; paolo.governa@unisi.it; 3 Department of Physical Sciences, Earth and Environment, University of Siena, Strada Laterina 8, 53100 Siena, Italy; marco.biagi@unisi.it

**Keywords:** mood disorders, L-theanine, excitotoxicity, neuroprotection, endocannabinoid system, CB1, flumazenil, anxiety

## Abstract

Mood disorders represent one of the most prevalent and costly psychiatric diseases worldwide. The current therapies are generally characterized by several well-known side effects which limit their prolonged use. The use of herbal medicine for the management of several psychiatric conditions is becoming more established, as it is considered a safer support to conventional pharmacotherapy. The aim of this study was to investigate the possible anxiolytic and antidepressant activity of a fixed combination of L-theanine, *Magnolia officinalis*, and *Melissa officinalis* (TMM) in an attempt to evaluate how the multiple modulations of different physiological systems may contribute to reducing mood disorders. TMM showed an anxiolytic-like and antidepressant-like activity in vivo, which was related to a neuroprotective effect in an in vitro model of excitotoxicity. The effect of TMM was not altered by the presence of flumazenil, thus suggesting a non-benzodiazepine-like mechanism of action. On the contrary, a significant reduction in the effect was observed in animals and neuronal cells co-treated with AM251, a cannabinoid receptor type 1 (CB1) antagonist, suggesting that the endocannabinoid system may be involved in the TMM mechanism of action. In conclusion, TMM may represent a useful and safe candidate for the management of mood disorders with an innovative mechanism of action, particularly as an adjuvant to conventional therapies.

## 1. Introduction

Mood disorders are a group of psychiatric illnesses representing the most prevalent and costly brain diseases worldwide [1]. The World Health Organization (WHO) estimates that 350 million people are affected by depression and anxiety, and this represents a huge impact in terms of economic cost and considering the negative impact on suffers’ quality of life [2]. The conventional management of mood disorders is based on pharmacotherapy and psychotherapy. However, the commonly used drugs are generally characterized by several well-known side effects which limit their prolonged use [3,4].

In recent years, the use of herbal medicine in the management of several psychiatric conditions has become more established, as it is considered a safer support to conventional pharmacotherapy [5]. Indeed, WHO and the European Medicines Agency describes several herbal medicinal products for their effectiveness in the management of different mood disorders that possess different mechanisms of action (i.e., anxyolitic: *Valeriana officinalis* L., *Passiflora incarnata* L., *Lavandula angustifolia* Mill.; antidepressant: *Hypericum perforatum* L.; adaptogens: *Rhodiola rosea* L., *Panax ginseng* C.A. Meyer) [6,7]. Recently, research efforts have been focused on the search for other herbal species which could enlarge the therapeutic approach.

Over the centuries, green tea (*Camellia sinensis* (L.) Kuntze) has been particularly used in Traditional Chinese Medicine for its sedative effect on subjects with mood disorder [8]. Particularly, L-theanine (TEA) is a water-soluble amino acid extracted from the leaves of *C. sinensis*, which has historically been used as a relaxing agent [9]. Its sedative effect seems to be related to the modulation of a wide range of neurotransmitters, and, in particular, to a reduction in glutamate transmission [10,11]. The glutamatergic tone is thought to act as a possible cofactor in increasing stress-related disorders such as anxiety [12]. Nowadays, TEA is receiving considerable interest in investigating its possible effect on cognition, mood, and brain function in humans [9].

The endocannabinoid system is an important physiological system able to influence and modulate several processes within the central nervous system [13], including mood disorders. Indeed, in the case of hyper-excitability, the activation of cannabinoid receptor type 1 (CB1) produces a reduction in the glutamatergic tone in order to maintain a normal synaptic activity [14,15]. *Magnolia officinalis* Rehder & E.H. Wilson plays an important role in traditional Chinese and Japanese herbal medicine, particularly for the treatment of mood disorders [16]. Moreover, the use of *Magnolia officinalis* has been described in the WHO monograph [17]. Honokiol, a neo-lignan extracted from the bark of *Magnolia officinalis*, shares a structural similarity with some cannabinoid receptors ligands and has been demonstrated to act as an agonist of CB1 [18].

Gamma aminobutyric acid (GABA) is the primary inhibitory neurotransmitter known to counteract the hyper-excitability effect of glutamate, and for this reason the GABA system represents an important target of anxiolytic drugs [19]. *Melissa officinalis* L. has been used in the treatment of anxiety and depression also in western medicine [20]. The main constituent, rosmarinic acid, has been shown to possess a GABA-transaminase inhibitory activity and beneficial effect on mood disorder modulation [21,22,23].

The aim of this study is to investigate the possible anxiolytic and anti-depressant activity of a fixed combination of TEA, a standardized *Magnolia officinalis* extract enriched in honokiol, and a standardized *Melissa officinalis* extract enriched in rosmarinic acid in order to evaluate how the multiple modulations of different physiological systems may contribute to reducing mood disorders.

## 2. Materials and Methods

### 2.1. Animals

Experiments were performed on male CD1 mice (weight: 22–24 g; age: 5–7 weeks, Envigo, Varese, Italy). The mice were randomly housed in standard cages and kept in a room at 23 ± 1 °C with a 12-h light/dark cycle with the light on at 7 a.m. Food (standard laboratory diet) and tap water were available ad libitum. The cages were placed in the experimental room 24 h before behavioral testing for acclimatization. All the tests were conducted during the light phase. The experimental protocol was approved by the Institution’s Animal Care and Research Ethics Committee (University of Florence, Italy) under license from the Italian Department of Health (54/2014-B). The mice were treated in accordance with the relevant European Union (Directive 2010/63/EU, the council of 22 September 2010 on the protection of animals used for scientific purposes) and international regulations (Guide for the Care and Use of Laboratory Animals, US National Research Council, 2011). All the studies involving animals are reported in accordance with the ARRIVE guidelines for experiments involving animals [24]. The experimental protocol was designed to minimize the number of animals used and their suffering.

The number of animals per experiment was based on a power analysis [25]. For behavioral assays, 8 animals per group were used to have a probability of 86%, at which the study detects a difference between groups (0.05 significance level). G power software was used to calculate the sample size.

### 2.2. Chemical and Drug Administration

Mice were randomly assigned to each treatment group by a researcher other than the operator. The fixed combination (TMM) of TEA (Giellepi Spa, Milan, Italy), *Melissa officinalis* leaf extract (MLE, extraction solvent: ethanol 50% *v*/*v*, standardized to contain 2% rosmarinic acid, MB Med, Rivalta di Torino, Italy), and *Magnolia officinalis* bark extract (MOE, extraction solvent: ethanol 96% *v*/*v*, standardized to contain 40% honokiol, Naturex Inc., South Hackensack, NJ, USA) was formulated to contain 25% TEA, 6.25% MLE, and 2.5% MOE. An amount of 1% sodium carboxymethyl cellulose (CMC, Sigma-Aldrich, Milan, Italy) was used as an inert excipient to solubilize the ingredients. This combination ratio was chosen on the basis of similar food supplements marketed in Italy. The TMM was administered p.o. 60 min before the test at the dose of 10 mg/kg for all experiments except for the dose–response curve, where doses of TMM ranging from 1 to 30 mg kg^−1^ p.o. were used. The control group received an equivalent volume of the vehicle. For comparison with the single components of the combination, the TEA, MLE, and MOE were administered at the concentration present in the active dose of TMM.

TMM, TEA, MLE, and MOE were dissolved in saline (0.9% NaCl) on the day of the experiment and administered at a volume of 10 mL/kg by gavage (p.o.). Each treatment was administered p.o. 60 min before the behavior test.

AM251 (4 mg/kg i.p.), flumazenil (FLU, 2 mg/kg i.p.) (Tocris, Bristol, UK), diazepam (DIAZ, 1 mg/kg i.p.), and amitriptyline (AMI, 10 mg/kg i.p) (Sigma Aldrich, Milan, Italy) were administered 30 min before the tests. The above-mentioned reference drugs were dissolved in a saline solution, except for AM251, which was dissolved in dimethyl sulfoxide/Tween 80/0.9% saline (1:1:18).

### 2.3. Locomotor Activity

#### 2.3.1. Rotarod Test

The possible onset of side effects on motor performance by each treatment was assessed by a rotarod test, as previously described [26]. The rotarod apparatus consisted of a 3 cm-diameter rod with a non-slippery surface at a rotation rate of 16 RPM. The rod (30 cm in length) was placed at a height of 15 cm from the base and divided into five equal sections by six disks. The animal was placed back on the rod immediately after falling, and the integrity of the motor coordination was assessed as the number of falls from the rod in 30 s. The test was performed 0, 15, 30, 45, 60, 90, 120, and 180 min after administration.

#### 2.3.2. Hole-Board Test

The spontaneous locomotor activity was evaluated using the hole-board test [26]. The apparatus consisted of an elevated arena (40 cm × 40 cm; 1 m above the floor) with 16 evenly spaced holes (3 cm in diameter; four lines of four holes each). The mice were placed individually on the center of the board and allowed to explore the plane freely for a period of 5 min each. The movements of the animal on the plane (spontaneous mobility) were automatically recorded by two photo beams, crossing the plane from the midpoint to the midpoint of opposite sides. Miniature photoelectric cells, placed in each of the 16 holes, recorded the head-dips in the holes by the mice. This head-dipping behavior represents the exploratory activity of the mice. The test was performed 60 min after oral administration.

#### 2.3.3. Hot Plate Test

The hot plate test was performed as previously described [26]. The mice were placed on a hot plate (Ugo Basile Biological Research Apparatus, Varese, Italy), with the temperature adjusted to 52.5 ± 0.1 °C. The reaction latencies (s) were measured with a stopwatch before (baseline latency) and after treatments. The time to the first sign of nociception (paw licking) was recorded and the mouse was immediately removed from the hot plate. An arbitrary cutoff period of 45 s was adopted to avoid damage to the paws. The test was performed 0, 15, 30, 45, 60, 90, 120 and 180 min after administration.

### 2.4. Anxiolytic-Like Activity

#### 2.4.1. Light-Dark Box

The light-dark box (LDB) apparatus (length 50 cm, width 20.5 cm, and height 19 cm) consisted of two equal acrylic compartments, one dark (black) and one illuminated by a 60 W bulb lamp (white). A dark insert (with black walls and lid, non-transparent for visible light) was used to divide the arena into two equal parts. The two compartments communicated by a small door (10 cm × 3.2 cm) at the floor level in the wall of the insert that allowed the animals to move freely from one compartment to another. Each mouse was released in the center of the light compartment with its head facing away from the door and allowed to explore the arena for 5 min. The behavioral parameters recorded were the time spent in the light chamber and the number of full-body transitions between chambers, since these have been previously described as a reflection of anxiety in this apparatus [27]. After testing, the animals were removed from the LDB and returned to their home cage in the colony room. After each test, the apparatus was cleaned with 70% ethanol to remove the olfactory cues and to allow it to dry before the next subject was tested. This test exploited the conflict between the animal’s tendency to explore a new environment and its fear of bright light.

#### 2.4.2. Marbles Test

The marble-burying behavioral test was performed as previously described [28]. All the experiments were conducted between 10:00 and 17:00. The mice were placed individually in clear plastic boxes (27 × 16 × 14 cm) containing 20 glass marbles (1 cm diameter) evenly spaced on sawdust 3 cm deep, without food and water. Each mouse was habituated to the behavior room for 30 min before being placed into the cage. The results of the test were expressed as the number of marbles buried for at least two-thirds within 30 min. The number of buried marbles is considered a measure of animal anxiety. The observer did not know which agent was being tested.

#### 2.4.3. Novelty Suppressed Feeding Test

The novelty suppressed feeding test (NSFT) test was performed as previously described [29]. The test was conducted in an uncovered plastic box (40 cm × 40 cm × 30 cm). The day before the test, the animals were acclimated for 10 min to the box and deprived of food overnight (12 h). On the day of the test, the mice was individually placed in a corner of the box, which contained a single pellet of food in the center. The latency to feed was recorded in 5 min. The observer did not know which agent was being tested.

### 2.5. Antidepressant-Like Activity

#### Tail Suspension Test

The tail suspension test (TST) was performed according to [30]. The mice were suspended from a plastic rod mounted 50 cm above the floor by adhesive tape placed to the upper middle of the tail. The time during which the mice remained immobile was measured with a stopwatch during a test period of 6 min. The mice were considered immobile when they hung passively and completely motionless, except for movements caused by respiration. Immobility was considered a depression-like behavior (behavioral despair) and was measured in the first 2 min of the test, when the animals react to the unavoidable stress, and in the last 4 min, when the behavioral despair is established.

### 2.6. Cell Culture

A human neuroblastoma cell line (SH-SY5Y, RRID:CVCL_0019), kindly donated by Prof. Lorenzo Corsi (University of Modena and Reggio Emilia, Italy), was cultured in DMEM (Sigma-Aldrich) and F12 Ham’s nutrients mixture (Sigma-Aldrich), containing 10% heat-inactivated FBS (Sigma-Aldrich), 1% L-glutamine (Sigma-Aldrich), and 1% penicillin-streptomycin solution (Sigma-Aldrich) until confluence (70–80%). The cells were grown in a humidified atmosphere with 5% CO_2_ at 37 °C. EDTA-trypsin solution (Sigma-Aldrich) was used for detaching the cells from flasks, and cell counting was performed using a hemocytometer by Trypan blue staining.

### 2.7. Cell Treatments

L-glutamate (GLU) was used to induce the excitotoxic stimulation of SH-SY5Y cells [31]. An 800 mM stock solution of monosodium glutamate (Sigma Aldrich, Milan) was prepared in sterile DMEM. Later, it was filtered and diluted in a complete medium for the cell treatments.

TMM (1 mg/mL), TEA (0.25 mg/mL), MOE (0.0625 mg/mL), and MLE (0.025 mg/mL) were dissolved in DMEM. The stock solution was then diluted in complete DMEM to reach the final working concentration, according to previously performed cell viability experiments (see supporting information).

The SH-SY5Y cells were pre-treated for 4 h with TMM, TEA, MOE, and MLE and then stimulated with glutamate 80 mM for 24 h. AM251 (Tocris) at an amount of 5 µM was administered 30 min before the pre-treatment with TMM, TEA, MOE, and MLE.

### 2.8. Cell Viability

Cell viability was performed using a Cell Counting Kit (CCK-8, Sigma-Aldrich) according to the manufacturer’s instructions. A total of 5 × 10^5^ cells/well were seeded into 96 multi-well plates and grown to confluence. The absorbance was measured at 450 nm using a MP96 microplate reader spectrophotometer (Safas, Monte Carlo, Principality of Monaco). The treatments were performed in six replicates in three independent experiments, and the cell viability was calculated by normalizing the values to the control’s mean.

### 2.9. Non-Competitive Sandwich ELISA Protocol for BDNF

A human brain-derived neurotrophic factor (BDNF) ELISA kit (Abcam, Milan, Italy) was used for the dosage of BDNF in human cell culture supernatant, according to the manufacturer’s instructions. SH-SY5Y cells (1.5 × 10^5^) were seeded in 200 µL of complete medium in 48-well culture plates and allowed to grow until confluence. The cells were pretreated with TMM, TEA, MLE, and MOE for 4 h and then stimulated with glutamate 80 mM for 24 h. The supernatants were collected and stored at −80 °C until analysis. The samples were analyzed in duplicate for a total of 3 independent experiments.

### 2.10. Data and Statistical Analysis

The data and statistical analysis in this study comply with the recommendations on experimental design and analysis in pharmacology [32]. The behavioral data are presented as means ± SEM. Eight mice per group were used. A two-way ANOVA followed by a Bonferroni post hoc was used for the statistical analysis. For the locomotor activity, the unpaired sample *t*-test was performed. For an in vitro analysis, the data were analyzed by a one-way ANOVA followed by a Tukey post hoc test. For each test, a value of *p* < 0.05 was considered significant. GraphPad Prism version 5.0 (GraphPad Software Inc., San Diego, CA, USA) was used for all the statistical analyses.

## 3. Results

### 3.1. Anxiolytic Effect of TMM

The LDB test aims to detect anxiety behavior in rodents, reflected by the avoidance of light areas and a decrease in explorative activity [33].

**Figure 1:**
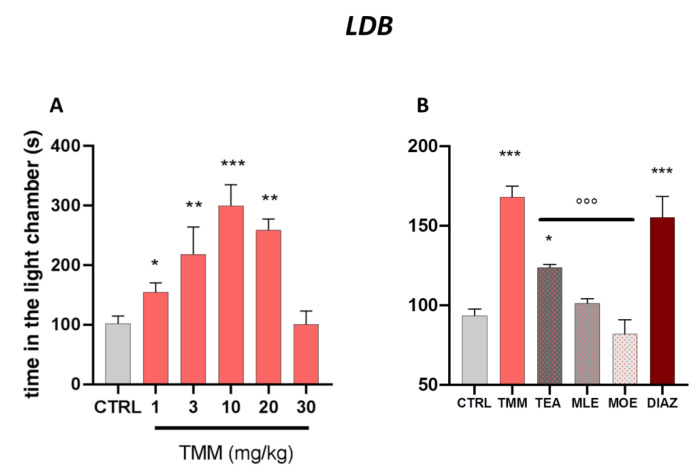
Dose–response curve obtained with the oral administration of different concentrations of TMM after 60 min in the light/dark (LDB) test (**A**). Comparison of the effect of TMM (10 mg/kg), L-theanine (TEA) (2.5 mg/kg), *Melissa officinalis* leaf extract (MLE) (0.625 mg/kg), and *Magnolia officinalis* bark extract (MOE) (0.25 mg/kg) in the L/D test (**B**). Diazepam (DIAZ) was used as a positive control. *** *p*< 0.001, ** *p*< 0.01, * *p* < 0.05 vs. CTRL; °°° *p* < 0.001 vs. TMM.

As reported in Figure 1A, TMM increased the time spent in the light chamber. The tice treated with TMM took less time to leave the dark compartment and spent significantly more time in the light chamber, thus showing an anxiolytic-like effect. The effect appeared 60 min after administration already with the dose of 1 mg/kg and the maximum effect was obtained with 10 mg/kg. Interestingly, at the dose of 30 mg/kg the effect disappeared, showing a biphasic bell-shaped effect.

TMM (10 mg/kg) resulted to be more effective than its constituents alone, used at the concentration present in the active dose of TMM (Figure 1B). Indeed, TEA (2.5 mg/kg) was the only component of TMM able to induce a significant increase in time spent in the light chamber compared to the control group, even if with a lower intensity than TMM. No significant effects were observed after the administration of MLE (0.625 mg/kg) and MOE (0.25 mg/kg).

The anxiolytic effectiveness of TMM in terms of time spent in the light chamber was comparable to that obtained with DIAZ, which was used as a reference drug.

**Figure 2:**
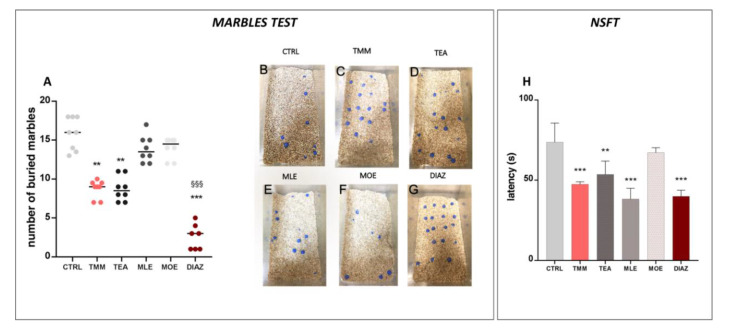
Effects of TMM (10 mg/kg), TEA (2.5 mg/kg), MLE (0.625 mg/kg), and MOE (0.25 mg/kg) in the marbles test (**A**), with representative images (**B**–**G**) and novelty suppressed feeding test (NSFT) (**H**). DIAZ was used as a positive control. *** *p* < 0.001, ** *p* < 0.01 vs. CTRL; ^§§§^ *p <* 0.001 vs. TMM.

In the marbles test, the number of marbles buried is an indicator of the emotional state of the mice. The higher is the number of buried marbles, the higher the anxiety-like behavior [28]. The control mice buried almost all of the marbles present in the box (79.06%; Figure 2A,B), while the TMM (Figure 2A,C)-treated mice buried significantly fewer (45.00%). An examination of the single components showed that TEA produced an anxiolytic-like behavior comparable to TMM (43.75%) (Figure 2A,D), whereas no significant effects were observed for MLE (68.00%; Figure 2A,E) and MOE (70.00%; Figure 2A,F). In this test, the effect induced by DIAZ was higher than TMM; indeed, the percentage of marbles buried was 12.85% (Figure 2A,G). In the NSFT, a reduced latency to interact with the food pellet is considered an anxiolytic effect [34]. As reported in the Figure 2H, TMM reduced the latency to interact with food in comparison to the control group (−35.64%). TEA also showed a significant reduction in the latency (−27.20%), similarly to MLE, which was able to reduce significantly the latency by approximately 48.10% compared to the control group. All of the substances described above possess an effect comparable to DIAZ, which reduces the latency of about 45.00% compared to the control group. Otherwise, no significant effects were observed with MOE (−8.81%).

### 3.2. Antidepressant-Like Activity in a Depressant-Like Paradigm

The effect of TMM in a depressant-like behavior task was investigated using the TST. The immobility time spent by the animals which was provoked by the unavoidable stress of being suspended by their tail is considered a depressive-like behavior [33]. The behavioral despair is established in the last 4 min of the test, in which the animal possesses an antidepressant-like phenotype.

**Figure 3:**
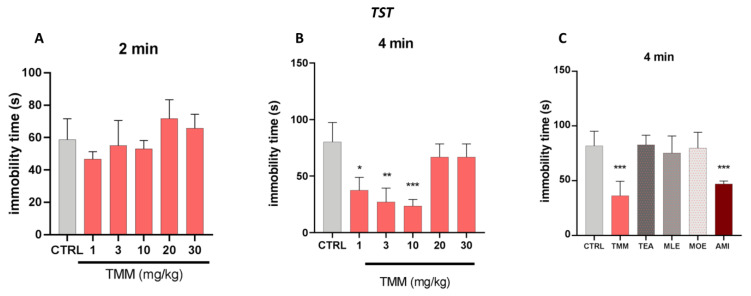
Evaluation of the antidepressant activity of TMM and its constituents. Dose–response curve obtained with different concentrations of TMM in the tail suspension test (TST), in the first 2 min (**A**) and in the last 4 min (**B**). Effect of TMM (10 mg/kg), TEA (2.5 mg/kg), MLE (0.625 mg/kg), and MOE (0.25 mg/kg) in the TST in the last 4 min (**C**). Amitriptyline (AMI) was used as an antidepressant reference drug. *** *p* < 0.001, ** *p* < 0.01, * *p* < 0.05 vs. CTRL.

No significant effects were observed in the first 2 min of the experiments (Figure 3A). TMM induced a significant reduction in the immobility time compared to the untreated mice at 1 (−56.47%), 3 (−68.46%), and 10 (72.53%) mg/kg, with 10 mg/kg being the most effective dose (Figure 3B). Similarly to the LDB results, no significant effects were observed at higher doses. The antidepressant-like effect of TMM was comparable to that obtained with AMI (−42.54%), used as a positive control. Interestingly, whereas the immobility time of mice treated with TMM resulted in being significantly lower than that of control group, no significant effect was observed in mice treated with TEA, MLE, and MOE (+1.16%, −7.90%, −2.50%; Figure 3C).

### 3.3. Lack of Impairment of Locomotor Behaviour and Hypersensibilization to Thermal Stimulus

The mice treated with TMM 10 mg/kg did not show any alteration in their gross behavior. The rotarod test and the hole-board test were used to evaluate the spontaneous mobility and exploratory activity of treated mice.

**Figure 4:**
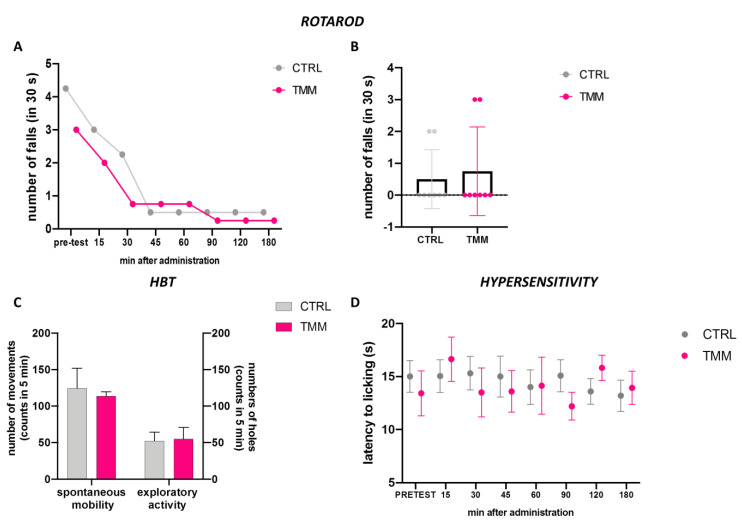
Lack of impairment of motor coordination observed with the rotarod test: 3 h time course (**A**) and 60 min after p.o. (**B**). Spontaneous mobility and exploratory activity measured by the hole board test 60 min after p.o. (**C**). Evaluation of the hypersensitivity by hot plate test from 0 to 180 min after p.o. (**D**).

The rotarod test analysis indicated that animals treated with TMM 10 did not show impaired motor coordination. Indeed, TMM (10 mg/kg)-treated mice showed a similar trend compared to the control group (Figure 4A,B). In addition, the spontaneous mobility and exploratory activity were unaltered by TMM administration compared to the control mice (Figure 4C). The alteration in glutamatergic transmission is involved in central sensitization, which is associated with chronic pain [35]. TMM did not alter the sensitivity to thermal stimuli compared to the control group (Figure 4D).

### 3.4. Neuroprotective Effect of TMM on SH-SY5Y Neuronal Cells

In order to evaluate the neuroprotective activity of TMM and its components, an in vitro excitotoxicity model was set up by stimulating SH-SY5Y neuronal cells with increasing concentrations of GLU for 24 h. GLU stimulation significantly reduces cell viability in a dose-dependent manner, with the concentration of 80 mM reducing cell viability by 25%, compared to the untreated control (Figure S1A). Moreover, this effect seems to be related to a reduction in the cell metabolic activity rather than a cytotoxic effect, as the extracellular release of lactate dehydrogenase (LDH) was not altered by GLU 80 mM (Figure S1B). The neuroprotective effect of TMM (1 µg/mL), TEA (0.25 µg/mL), MLE (0.0625 µg/mL), and MOE (0.025 µg/mL) was tested by pre-treating the cells for 4 h before the GLU 80 mM stimulation.

**Figure 5:**
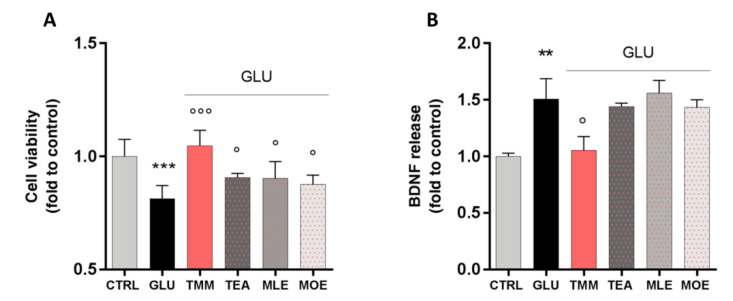
Neuroprotective effect induced by TMM in L-glutamate (GLU)-stimulated SH-SY5Y cells compared to TEA, MLE, and MOE (**A**). Modulation of brain-derived neurotrophic factor (BDNF) release after GLU stimulation by TMM, TEA, MLE, and MOE (**B**). *** *p* < 0.001, ** *p < 0.01* vs. CTRL; °°° *p < 0.001, ° p < 0.05* vs. GLU.

TMM was able to counteract the excitotoxic effect by preventing the reduction in cell viability induced by GLU. Even if with a lower potency, a similar trend was observed for TEA, MLE, and MOE (Figure 5A). Thus, the effect of TMM seems to be related to the sum of each components, rather than to only one of them.

Recently, a correlation between the dysregulation of brain-derived neurotrophic factor (BDNF) in the CNS and mood disorders has been reported [36]. In our model, the excitotoxic effect induced by GLU produced a marked increase in BDNF release by neuronal cells, which was significantly reduced by the pretreatment with TMM. On the contrary, the use of TEA, MLE, and MOE alone had no significant effect on the BDNF release (Figure 5B).

### 3.5. Involvement of Endocannabinoid System in the Final Effect of TMM

In order to better investigate the implication of the endocannabinoid system in the anxiolytic effect of TMM, a pretreatment with the CB1 antagonist AM251 was administered before the L/D box, leading to a reduction in the effectiveness of TMM 10 mg/kg.

**Figure 6:**
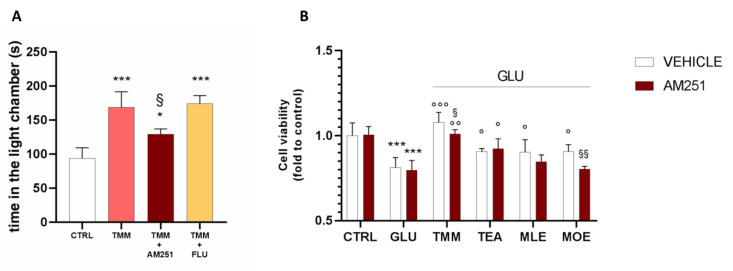
Comparison of the anxiolytic effect of TMM in the presence and absence of AM251 and FLU. Vs. CTRL; ^§^ *p* < 0.05 vs. TMM; * *p* < 0.05 vs. CTRL (**A**). Comparison of the effect on the SH-SY5Y cell viability in the presence and absence of AM251. *** *p* < 0.001 vs. CTRL; °°° *p* < 0.001 vs. GLU; °° *p* < 0.01 vs. GLU; ° *p* < 0.05 vs. GLU; ^§§^ *p* < 0.01 vs. vehicle; ^§^ *p* < 0.05 vs. vehicle (**B**).

In order to deepen the mechanism of action of TMM, a pretreatment with FLU (2 mg/kg), a GABA A benzodiazepine-binding site antagonist, was administered. The co-administration of TMM with FLU did not impair the TMM effectiveness, thus excluding a benzodiazepine-like mechanism of action (Figure 6A). The involvement of the endocannabinoid system has been confirmed also in vitro. Indeed, the pretreatment with AM251 reduced the neuroprotective effect of TMM (Figure 6B). The administration of the CB1 antagonist did not altered the effect of TEA and MLE; however, it induced a reduction in the neuroprotective activity of MOE.

## 4. Discussion

Mood disorders are the most common and prevalent behavioral disorders and can result in significant impairment of patients’ quality of life [37]. Anxiety disorders are among the most prevalent psychiatric disorders worldwide, with their pharmacological treatments often being unsatisfying. The development of alternative therapies for the management of these pathological conditions, hence, is of much interest. In recent years, the role of herbal medicine in the treatment of mood disorders has become more established [5]. From this perspective, we have focused this study on the effect of a fixed combination of TEA, MOE, and MLE in order to evaluate how the multiple modulation of different physiological systems, such as the glutamatergic, GABA-ergic, and cannabinoid, may cooperate in reducing the symptoms associated with mood disorders. In our experiments, we demonstrated that a single oral administration of TMM was able to reduce anxiety behavior compared to the control group after 60 min, with an efficacy comparable to that of diazepam, which was used as a benzodiazepine positive control. The TMM efficacy was higher than its components alone, highlighting a synergistic mechanism of action. We also found that the dose-effect relationship of TMM was biphasic, showing a bell-shaped effect. Natural products have been frequently shown to have a biphasic pharmacological effect, both in vitro and in vivo [38,39,40]. In particular, we previously reported that a standardized *Hypericum perforatum* L. extract, which is a well-known anti-depressant herbal drug, can exert an anti-nociceptive effect in mice models of neuropathic pain at lower doses compared to the one used for treating depression, with a mechanism of action involving the opioid system. This effect was not observed when administering higher doses, thus confirming the biphasic trend [41].

Anxiety and depression are often comorbid conditions [42]. WHO declared that 320 million people suffer from depression, and the number is increased if “low mood” subjects are included. Antidepressant and anxiolytics drugs often result in being ineffective [43,44] and/or cause intolerable side effects [45]. Thus, a drug candidate claimed to be safe and effective for both anxiety and depression would represent the ideal therapy. TMM showed an interesting antidepressant-like activity in TST compared to amitriptyline (a conventional tricyclic antidepressant drug), while no significant effects were observed administering its components alone. TEA has been reported to possess antidepressant effects in humans and animal studies. Indeed, Hidese and co-workers showed that 8 weeks administration of TEA (250 mg/day) had beneficial effects on depressive symptoms, anxiety, sleep disorders, and cognitive impairment in patients with mild major depressive disorders [46]. The antidepressant-like activity of repeated TEA (0.4–20 mg/kg) administration was also confirmed in vivo using the open-field test, the forced swim test, the elevated plus-maze test, the prepulse inhibition of acoustic startle, the tail suspension test,, and the reserpine test [47,48]. On the contrary, in our study we were not able to observe an antidepressant-like effect for TEA alone. This negative result could be due to differences in the doses, administration route, and schedule compared to the other studies. Our results suggest an additive effect between TEA, MLE and MOE and showed the peculiar dual activity of TMM as an antidepressant and anxiolytic agent. Moreover, differently from other conventional drugs [49], TMM did not induce sedation or alteration in motor coordination, thus representing a clinical advantage for long-term therapies.

Recently, it was found that 4 weeks of administration of TEA (200 mg/day) was also able to reduce stress-related symptoms and improve cognitive functions in healthy adults [50]. Moreover, TEA was reported to possess beneficial effects in the treatment of anxiety and sleep disturbance [51,52,53]. Its activity seems to involve the inhibition of presynaptic glutamate release [11] and antagonism at the N-metyl-D-aspartate (NMDA) and α-amino-3-hydroxy-5-methyl-4-isoxazolepropionic acid (AMPA) glutamate receptors [54], leading to a reduction in over-activated glutamatergic neurotransmission. Interestingly, TEA (250 mg/day for 8 weeks) was reported to ameliorate positive symptoms and sleep quality in patients with schizophrenia by stabilizing the glutamatergic concentration in the brain [55]. The modulation of glutamate levels in brain by TEA was further confirmed in vivo by Ogawa and collaborators, who reported that L-theanine enhances anxiolytic effects, reducing the cerebrospinal fluid glutamate concentration [56].

The glutamatergic system has been demonstrated to possess a key role in the pathogenesis of anxiety [12]. Indeed, the administration of NMDA and non-NMDA glutamate receptors antagonists into the basolateral amygdala was reported to reduce anxiety in animal models [57]. Moreover, the balance between GABA receptor-mediated inhibition and glutamate receptor-mediated excitation regulates the behavioral and physiological responses associated with anxiety [58]. *Melissa officinalis* has been shown to possess anxiolytic and anti-depressant activity by modulating GABA transmission in animal models [23,59,60] and humans [61]. Particularly, Awad and co-workers demonstrated that rosmarinic acid was the main responsible for the anxiolytic effect of *Melissa officinalis* [23].

Interestingly, the cross-talk between GABA-ergic and glutamatergic neurotransmission has been reported to be mediated by the CB1 receptor [62]. Honokiol, a lignan extracted from *Magnolia officinalis*, has been shown to act as a potent full agonist at the CB1 receptor [18], and studies conducted with *Magnolia officinalis* bark extracts have reported possible anxiolytic activity without significant side effects [63,64].

In order to deeply investigate the synergism between TMM components, we evaluated the neuroprotective effect of TMM and its single components in an in vitro model of excitotoxicity. GLU is the major excitatory neurotransmitter in the brain and it modulates a plethora of functions, such as memory, pain motor function, and mood tone [65]. Indeed, the glutamate system has been successfully targeted in both pre-clinical and clinical studies, providing a good efficacy in the treatment of anxiety disorders [66]. In vitro models of GLU-induced neuronal damage have been widely used to test the mechanism of action of well-known anxiolytic drugs or novel neuroprotective candidates [67,68,69]. By inducing excitotoxicity in SH-SY5Y neuronal cells with GLU, we demonstrated the neuroprotective effect of TMM. A weaker effect was obtained with its components alone. BDNF is a neurotrophin which is pivotal for the survival, growth, and maintenance of neurons in key brain circuits involved in emotional and cognitive function. Indeed, the alteration of the normal BDNF level is strongly linked with mood disorder manifestation [70,71,72]. The glutamate receptor-mediated BDNF release a main evidence of a cooperative interaction between BDNF and GLU [73,74]. Indeed, the excitotoxic cascade in neurons stimulates the release of BDNF as a defense mechanism against the oxidative stress produced by the hyper-activation of the glutamate receptor [75,76]. The pre-treatment with TMM completely prevented the GLU-increased release of BDNF in SH-SY5Y, highlighting a protective effect against excitotoxicity. On the contrary, TEA, MLE, and MOE alone were not able to counteract this effect. Benzodiazepines are characterized by a reduced patient compliance because of their well-known side effects [77]. In order to exclude a benzodiazepine-like mechanism of action of TMM, we treated mice in the presence of FLU, a well-known benzodiazepine antagonist [78]. The effect of TMM was not altered by the presence of FLU, thus suggesting a non-benzodiazepine-like mechanism of action. On the contrary, a significant reduction in the effect was observed in animals and neuronal cells co-treated with AM251, a CB1 antagonist [79], and TMM. Confirming the reliability of our model, the MOE effect was significantly reduced by the CB1 antagonist. This is consistent with the reported CB1 agonist activity of MOE constituents in neuronal cells [18].

## 5. Conclusions

The results obtained in this study highlighted that the co-administration of a GABA-modulating agent (MLE), a modulator of the glutamate receptor (TEA), and a modulator of the CB1 receptor (MOE) could be suggested as a novel and interesting anxiolytic and antidepressant strategy, with a neuroprotective effect against excitotoxicity and being devoid of the typical side effects of anxiolytic drugs. TMM may represent a useful and safe approach for the management of mood disorders in pre-clinical forms or in case of low mood, as well as an adjuvant to conventional therapies.

## Supplementary Materials

The following are available online at https://www.mdpi.com/2072-6643/12/6/1803/s1, Figure S1: Effect of increasing concentration of GLU on SH-SY5Y cell viability, evaluated by CCK-8 kit (Sigma-Aldrich, Milan, Italy) (A). The cytotoxic potential of the stimulus was excluded by measuring the lactate dehydrogenase (LDH) release, using LDH activity assay (Sigma-Aldrich, Milan, Italy).
