# Supplementary material for: Novel Therapeutic Approach for the Management of Mood Disorders: In Vivo and In Vitro Effect of a Combination of L-Theanine, Melissa officinalis L. and Magnolia officinalis Rehder & E.H. Wilson
Source: Nutrients. 2020 Jun 17;12(6):1803. doi: 10.3390/nu12061803 (PMC7353338; doi:10.3390/nu12061803)
Supplement: Supplementary file 1 [file nutrients-12-01803-s001.pdf]

Novel therapeutic approach for the management of mood disorders: *in vivo* and *in vitro* effect of a combination of L-theanine, *Melissa officinalis* L. and *Magnolia officinalis* Rehder & E.H. Wilson.

Vittoria Borgonetti [1,†], Paolo Governa [2,†], Marco Biagi [3] and Nicoletta Galeotti [1,*]

[1] Department of Neuroscience, Psychology, Drug Research and Child Health (NEUROFARBA), Section of Pharmacology, University of Florence, Viale G. Pieraccini 6, 50139 Florence, Italy; vittoria.borgonetti@unifi.it, nicoletta.galeotti@unifi.it

[2] Department of Biotechnology, Chemistry and Pharmacy - Department of Excellence 2018-2022, University of Siena, Via Aldo Moro 2, 53100 Siena, Italy; paolo.governa@unisi.it

[3] Department of Physical Sciences, Earth and Environment, University of Siena, Strada Laterina 8, 53100 Siena, Italy; marco.biagi@unisi.it

* Correspondence: nicoletta.galeotti@unifi.it; Tel.: +39-055-275-8391

† These authors contributed equally to this work

Supplementary material

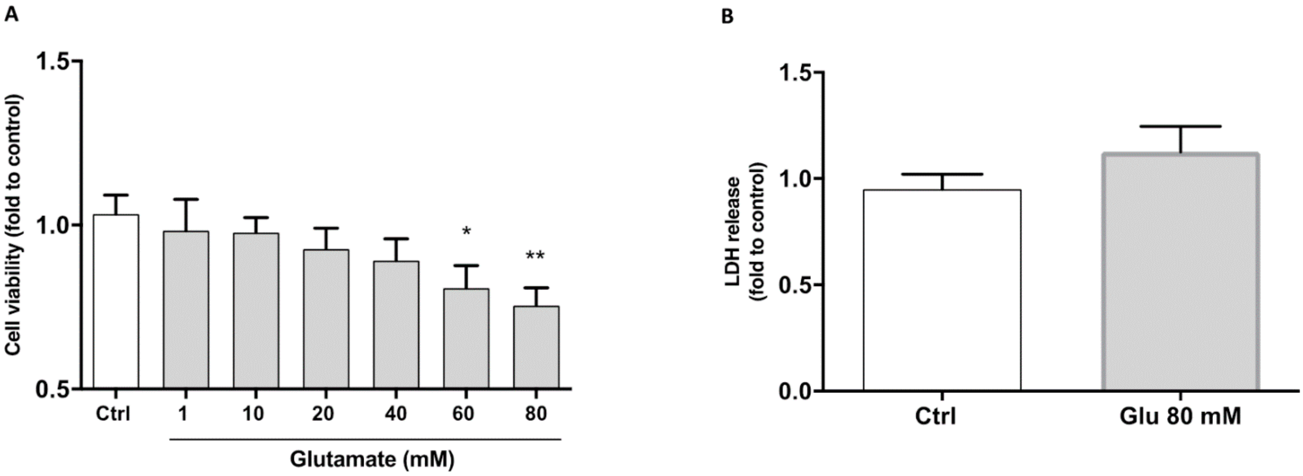

Figure S1. Effect of increasing concentration of GLU on SH-SY5Y cell viability, evaluated by CCK-8 kit (Sigma-Aldrich, Milan, Italy) (A). The cytotoxic potential of the stimulus was excluded by measuring the lactate dehydrogenase (LDH) release, using LDH activity assay (Sigma-Aldrich).
